# Digital, Personalized Clinical Trials Among Older Adults, Lessons Learned From the COVID-19 Pandemic, and Directions for the Future: Aggregated Feasibility Data From Three Trials Among Older Adults

**DOI:** 10.2196/54629

**Published:** 2025-04-16

**Authors:** Lindsay Arader, Danielle Miller, Alexandra Perrin, Frank Vicari, Ciaran P Friel, Elizabeth A Vrany, Ashley M Goodwin, Mark Butler

**Affiliations:** 1 Institute of Health System Science, Feinstein Institutes for Medical Research, Northwell Health New Hyde Park, NY United States

**Keywords:** older adults, personalized, digital, clinical trial, remote, physical activity, back pain, pain, COVID-19, clinical trials, pandemic, chronic health, digital intervention, Fitbits, Fitbit, wearable, wearables, exercise, gerontology, geriatric, geriatrics, old, older, older people, aging, aged, mobile phone

## Abstract

**Background:**

The COVID-19 pandemic was extremely disruptive to clinical practice and research. Given older adults’ increased likelihood of chronic health concerns, limited resources, and greater risk for adverse outcomes of COVID-19, access to research participation during this time was critical, particularly to interventions that may impact health conditions or behaviors. Fortunately, the implementation of personalized, digital research trials during the pandemic allowed for research and intervention delivery for older adults to continue remotely, resulting in feasibility findings that can benefit researchers, practitioners, and the broader older adult population.

**Objective:**

This study discusses 3 digital, remote, and personalized intervention trials implemented during the pandemic to increase physical activity (2 trials) or to reduce back pain (1 trial).

**Methods:**

We identified measures used for all 3 trials including Fitbit activity monitor use and self-reported participant satisfaction. Participant levels of Fitbit activity monitor use and satisfaction ratings of the digital trials were compared between younger (younger than 55 years) and older adults (older than 55 years). Differences between these cohorts were analyzed using chi-square tests for categorical outcomes and 2-tailed independent-sample *t* tests for continuous outcomes.

**Results:**

Across the 3 trials, the majority of participants reported high satisfaction with the usability of the trials’ digital systems including SMS text message interventions and surveys (≥62% satisfied) and the use of wearable devices such as Fitbits (≥81% satisfied). In addition, the use of the Fitbit device was shown to be feasible, as older adults across all trials wore their Fitbits for the majority of the day (mean 20.3, SD 3.6 hours). Furthermore, consistent Fitbit wear was common; 100% of participants older than 55 years wore their Fitbit an average of 10 or more hours per day. These trials highlight that digital, remote intervention delivery may be successfully implemented among older adults by way of personalized trials. Across the 3 digital interventions, feasibility and acceptability were high among older adults, and comparable to younger adults.

**Conclusions:**

Given the success of the current trials amid pandemic restrictions, we argue that these trials serve as a useful framework to aid in designing personalized, digital, remote interventions in other areas of clinical care among older adults and in planning for future disruptions including new pandemics.

## Introduction

### Background

COVID-19 is ranked among the worst pandemics in global history [1], with impacts ranging beyond the death toll [2] to nearly all facets of society including general health care accessibility. In the United States, outpatient visits to ambulatory care clinics declined by 60% in the early months of the pandemic [3]. Among older adults specifically, there is evidence that about 35% of routine in-person physician visits were canceled in 2020 [4], raising concerns about older adults’ access to necessary health care services in the context of being at higher risk for medical comorbidities, mental health problems, and loneliness [5]. Following this decline, demand for digital clinical care increased as a means of enhancing the accessibility of support [6], and telemedicine interventions for COVID-19 were implemented to reduce the risk of transmission between patients and providers [7]. Though this new digital landscape increased access to care for many, it also imposed new barriers for the older adult population, such as contending with the inconvenience, stress, and digital literacy limitations that come with adapting to unfamiliar technology [5].

Participation in clinical trials also had worse experiences due to the effects of COVID-19. Between October 2019 and June 2020, there was a 30% decline in enrollment of participants across several key medical research areas, including cardiovascular health, oncology, and respiratory health [8]. Researchers also reported dropout rates for participants in their trials were as high as 49% [9]. Trial participation was especially difficult during COVID-19 for older adults given national guidance on limiting in-person interactions of and with vulnerable populations, such as older people [10]. By some estimates, older adults were underrepresented in research that occurred during the COVID-19 pandemic [11]. Disruptions to trial conduct, recruitment, and enrollment became so common that new guidelines were put in place to modify the traditional CONSORT (Consolidated Standards of Reporting Trials) [12] diagram to account for disruptions due to COVID-19. These new CONSERVE (CONSORT and SPIRIT [Standard Protocol Items: Recommendations for Interventional Trials] Extension for RCTs [randomized controlled trials] Revised in Extenuating Circumstances) guidelines helped researchers document how COVID-19 interfered with research conducted during the pandemic [13].

### Digital, Remote Clinical Trials Among Older Adults

Fortunately, advances in technology and innovations prior to and during the COVID-19 pandemic have improved the ability for interventions to be delivered remotely among older adults. Wearable devices used in remote health research have been shown to be effective in monitoring older adults with chronic conditions, and patients at risk for cardiovascular events, and in providing accurate assessment, behavior tracking, stress monitoring, diagnosis, disease management, biomarkers, and several other variables [14-16]. The types of outcomes that may be assessed remotely have expanded greatly, even allowing blood samples to be collected in the home by the patient and sent to labs for analysis [17-19]. Despite the presumed barriers to enrolling older adults in digital, remote clinical research, several trials conducted during the COVID-19 pandemic have succeeded in this pursuit, including a longitudinal brain aging study [20], a telemedicine initiative in a primary care setting [21], telehealth delivery of music therapy services [22], and a digital group intervention addressing worry and social isolation [23]. Across these studies, research teams used participant retention strategies such as regular check-ins (via SMS text message, phone, and email), digital training of participants in the use of study devices and procedures (such as videos), and feedback sessions to promote engagement and digital literacy. The success of these trials paves the way for further investigation into clinical trials tailored to older adults. The personalized trials framework is a unique pathway to achieve this.

### The Rationale for Digital, Remote Personalized Trials Among Older Adults

Personalized trials are trials that focus on or are tailored to a single person [24]. A special case is the personalized N-of-1 trial, which is a randomized clinical trial conducted at the level of the individual [25]. Personalized trials have several advantages. First, they can identify optimal care for individuals that typically would not meet the criteria for clinical trials, due to, for example, multimorbidities or rare diseases [26,27]. In addition, they can test the effectiveness of combining different interventions or tailoring treatment for one person [27]. Research has shown that personalized care not only leads to physical and psychological improvements but it also enhances an individual’s capacity to manage their condition [28]. Among older adults, the benefits of personalization are particularly important as they are a population that is frequently excluded from clinical research [29]. Personalized trials conducted among older adult cohorts have demonstrated improvement in outcomes across a range of conditions, including chronic pain [30], depression [31], physical activity [32], cancer [33], and the experience of palliative care [34].

While a patient-focused structure in a personalized trial has always been appealing, the uptake for such trials in practice and research has been low [35]. This is largely because personalized designs require effort to design and refine an intervention that is tailored for each individual participant. However, with the development and integration of new technologies as a result of the COVID-19 pandemic, it is now more feasible than ever to conduct personalized trials.

Fortunately, researchers have recently begun to explore the feasibility of personalized trials as a method for conducting remote research. During the pandemic, researchers at the Feinstein Institutes for Medical Research at Northwell Health conducted 3 remote, personalized trials with all processes from recruitment through outcome assessment occurring remotely, including intervention delivery (either via smartphone app or in the participant’s home without in-person contact with research study personnel). These trials focused on increased walking among older adults, developing a walking habit among older adults, and reducing chronic lower back pain (CLBP). Though the trial focused on treating CLBP symptoms and did not specifically recruit older adults, there was a large population of individuals in the trial who were older than 55 years.

Notably, all trials discussed in this study were designed prior to March 2020. Due to the unexpected nature of the COVID-19 pandemic, the remote, digital trial designs provided an opportunity to retroactively evaluate participant satisfaction with the trials in the context of global restrictions to necessary care.

In this study, we examine results from 3 remote, personalized, digital trials to identify their feasibility among older and younger adults in terms of wearable device use and participant satisfaction. The goals of this analysis are to determine whether older adults are adherent to using wearable devices and satisfied with several aspects of digital, personalized trials including digital study onboarding, digital survey assessment, using wearable devices, and personalized trial design. We hope that findings from these trials that used digital recruitment and intervention delivery, aligning with the restrictions during the COVID-19 pandemic, may support the use of digital, personalized research designs among older adults in the future.

## Methods

### Study Design

This study uses Fitbit data and satisfaction measures from 3 different personalized trials to identify the potential feasibility and acceptability of digital personalized research among older adults. In this study, “feasibility” and “acceptability” are operationalized as participant’s adherence to trial expectations and reported satisfaction with trial components, respectively. Specific measures of adherence and satisfaction are described in the measures section. All 3 trials made use of digital recruitment during the COVID-19 pandemic, used remote intervention delivery via smartphone app or in the participant’s home without in-person contact with research study personnel, involved wearing a Fitbit activity tracker, and used SMS text message survey assessments. The eligibility criteria for each trial can be found in Multimedia Appendix 1.

### Study Design—Trial #1—Behavior Change Techniques to Improve Low-Intensity Physical Activity in Older Adults (NCT04967313)

The methods of this trial have been previously published in greater detail [36]. Briefly, this study was a series of 60 randomized, personalized trials examining the effects of 4 behavior change techniques (BCTs) to increase low-intensity walking among middle and older adults (aged 45 to 75 years). The 4 BCTs used were goal setting, action planning, self-monitoring of behavior, and feedback on behavior. Prior research has shown these BCTs to be effective in increasing physical activity among older adults [37]. Each personalized trial was comprised of a 2-week baseline period followed by an 8-week intervention period. During the intervention, participants received each of the BCTs individually delivered in four 2-week blocks. For example, one participant may have received a 2-week block of action planning, followed by 2 weeks of feedback, then 2 weeks of goal setting, and finally 2 weeks of self-monitoring. The order in which BCTs were delivered was randomized for each participant by the study statistician. The goal of the trial was to increase low-intensity walking by 2000 steps per day between the baseline and intervention periods. Steps were measured using a Fitbit activity monitor. Study recruitment was conducted between November 5, 2021, and May 5, 2022.

### Study Design—Trial #2—A Trial of Habit Formation Theory for Exercise in Older Adults (NCT04869644)

The methods of this trial have been previously published in greater detail [38]. In brief, this study was a series of 32 randomized, personalized trials evaluating the effects of 5 BCTs to enhance habitual, low-intensity walking among older adults (aged 45 to 75 years). The 5 BCTs used were goal setting, action planning, self-monitoring of behavior, behavioral practice or rehearsal, and habit formation. These BCTs have previously been associated with habitual formation theory [39] and with improving physical activity [40]. Each trial was comprised of a 2-week baseline period followed by a 10-week intervention period. During the intervention, participants received the 5 BCTs daily via SMS text messages that were timed based on the participant’s walking habits. For example, if participants planned to walk in the morning, the self-monitoring BCT (defined as monitoring one’s own behavior) would be delivered in the afternoon. The goal of the trial was to increase the automaticity of habitual daily walking. Steps were measured using a Fitbit activity monitor. Study recruitment was conducted between March 26, 2021, and February 1, 2022.

### Study Design—Trial #3—Personalized Trial for CLBP (NCT04203888)

The methods and results of this trial have been previously published in greater detail [41]. Briefly, this trial was a series of 57 randomized, personalized N-of-1 trials examining the effect of yoga and massage on symptoms of CLBP. Participants completed a 2-week baseline period and a 12-week intervention period (14 weeks total). During the intervention period, participants received six 2-week treatment blocks alternating between yoga, massage, and usual care. There were two treatment orders in the trial: (1) massage, yoga, usual care, usual care, yoga, and massage; and (2) usual care, yoga, massage, massage, yoga, and usual care. Across all 14 weeks, participants were encouraged to wear a Fitbit activity monitor, complete SMS text message ecological momentary assessments of pain, and complete additional measures via SMS text message surveys. The ecological momentary assessment of pain was selected as a key measure because it provides insight into a participant’s experiences using repeated real-time assessments of pain in a person’s typical environment [42]. Massage and yoga sessions were booked using Zeel, a commercially available platform, and delivered in the participant’s homes by trained providers. The primary goal of the trial was to identify the feasibility and acceptability of personalized trials for CLBP. The study recruitment occurred between November 20, 2019, and January 31, 2021. There was a pause in the study recruitment and intervention delivery between March 21, 2020, and August 3, 2020, to comply with the “New York State on PAUSE” executive order.

### Measures

#### Participant Characteristics

For each trial, we identified the proportion of older adults who participated in the trial. For the purposes of this analysis, we will be defining older adults as individuals who are 55 years or older. Though many definitions exist for what age defines an “older adult,” [43-45], we included individuals aged 55 years and older for the most expansive inclusion of all potential older adults. Furthermore, extending the age range for this study allows us to capture feasibility data from both Generation X and baby boomer participants; both of whom can be considered “digital immigrants” based on formative development prior to the widespread implementation of digital tools [46,47].

#### Fitbit Wearable Device Use

All trials used Fitbit activity monitors to track participant levels of physical activity. For the current analysis, we examined several metrics of adherence to the Fitbit devices used in each trial to determine the feasibility of device integration. First, we identified the mean and SD of daily Fitbit wear time for each trial and by age group in the trial (<55 vs ≥55 years old). We also identified the range in daily Fitbit wear time. We specifically identified the proportion of older and younger participants in each trial with an average of 10 or more hours of Fitbit wear time per day for the entire study duration as an indicator of sufficient adherence. We also calculated this proportion for participants <55 and ≥55 years old. Finally, we identified the proportion of individuals who had valid Fitbit wear days (defined as wear for ≥10 hours per day) for 80% or longer of the study duration. Many prior studies have used ≥10 hours per day as a criterion for a “valid wear day” [48-52]. As with other metrics, we identified this proportion for individuals <55 and ≥55 years old.

#### Acceptability

All trials administered satisfaction surveys after trial completion to assess trial acceptability. These satisfaction surveys are based on standard elements used in several previously conducted personalized trials [36,38,41,53-57] with modifications specific to each of the methods of each trial. In Trial #1, two separate satisfaction measures developed by the study team were administered. The first was a 9-item measure assessing satisfaction with trial elements, rated using a 4-point Likert scale from 0=not at all satisfied to 3=very satisfied. The second measure was an 11-item measure assessing attitudes toward elements of the personalized trial, rated on a 7-point Likert scale from 0=strongly disagree to 6=strongly agree. Similarly, 2 measures of satisfaction developed by the study team were administered in Trial #2. The first was a 9-item survey assessing satisfaction with the trial on a 4-point Likert scale from 0=not at all satisfied to 3=very satisfied. The second measure was a 9-item survey on specific elements of the personalized trial rated on a 7-point Likert scale from 0=strongly disagree to 6=strongly agree. Though the structure of these satisfaction measures was similar to measures used in Trial #1, the individual items were tailored to the designs of Trial #2. In Trial #3, participants received the System Usability Scale, a measure used to assess the usability of both clinical trials and other systems [58,59]. The System Usability Scale is a 10-item survey rated on a 5-point Likert scale from 1=strongly disagree to 5=strongly agree, focused on the personalized trials system as a whole. Participants also completed a 7-item survey developed by the study team and rated on a 5-point Likert scale from 1=strongly disagree to 5=strongly agree assessing attitudes about elements of the trial and another 8-item survey assessing satisfaction with the trial rated on a 5-point Likert scale from 1=not at all satisfied to 5=very satisfied.

For the purposes of this analysis, we focused on 6 specific dimensions of satisfaction with the personalized trials, which were consistently assessed across all 3 trials. First, we examined whether participants felt the Fitbit wearable activity tracker was easy to use. Second, we examined participant attitudes about the study onboarding process, which included the Fitbit device setup. Third, we examined participant satisfaction with the explanatory videos used in all 3 trials to describe the trial design and process. Fourth, we examined participant satisfaction with completing surveys and receiving SMS text message interventions via cellular phone. Fifth, we examined whether participants felt burdened by their engagement in the personalized trial. Finally, we examined whether participants would recommend their personalized trial to others. We calculated means and SDs for participant satisfaction ratings for each item on all 3 trials, as well as ratings for participants <55 versus ≥55 years old within each trial. As the use of Fitbit wearable activity trackers and SMS text message interventions or surveys was essential to the designs of all trials, we also reported the proportion of participants in each trial who were satisfied with these elements. For these proportions, satisfaction was defined as a response higher than the midpoint on the Likert scale (eg, a score of 4 or more on a 7-point Likert scale ranging from 0 to 6). Measures that were initially reverse-scored in the original study were recoded for ease of comparison between trials. To compare satisfaction levels between older and younger adults, we conducted 2-tailed, independent samples *t* tests for continuous variables and chi-square tests for categorical variables. For cases where the assumptions of chi-square tests may be invalid (eg, 0 participants were dissatisfied), we used Mann-Whitney *U* tests. Note that comparative analyses should be interpreted cautiously given relatively small sample sizes.

### Ethical Considerations

As this study used secondary analyses of freely available, deidentified data, no institutional board approval was required for this project.

## Results

### Participant Characteristics

Though not all participants in each of the trials were older adults, a significant proportion of participants in each trial were older than 55 years (Trial #1: n=29, 48%; Trial #2: n=22, 72%; Trial #3: n=14, 25%). The majority of participants in each of the 3 trials were female (Trial #1: n=55, 92%; Trial #2: n=27, 84%; Trial #3: n=42, 74%). Full participant characteristics for each trial can be found in Table 1.

**Table 1:** Sample Characteristics of 3 personalized trials among older adults.

		Trial #1—behavior change techniques to improve low-intensity physical activity in older adults (n=59), n (%)	Trial #2—a trial of habit formation theory for exercise in older adults (n=32), n (%)	Trial #3—personalized trial for chronic lower back pain (n=57), n (%)
Age (55 years or older)		29 (49)	22 (69)	14 (26)
**Sex**				
	Female	55 (92)	27 (84.4)	42 (74)
	Male	5 (8)	5 (16)	15 (26)
**Race**				
	American Indian or Alaskan Native	1 (1.7)	0 (0)	0 (0)
	Asian	4 (7)	3 (9)	11 (19)
	Black	5 (9)	2 (6)	6 (11)
	Mixed or more than one race	0 (0)	1 (3)	2 (4)
	Other or unknown or not reported	2 (3)	1 (3)	4 (7)
	White	47 (80)	25 (78)	34 (60)
**Ethnicity**				
	Hispanic	3 (5)	5 (16)	8 (14)
	Non-Hispanic	56 (95)	27 (84)	49 (86)

### Fitbit Wearable Device Adherence

Across all trials, the mean daily Fitbit wear time was high at approximately 20.3 hours (mean, 1217.47, SD 217.53 minutes). Mean Fitbit wear time was also high among individuals aged 55 years and older at approximately 20.7 hours (mean 1240.94, SD 200.43 minutes). The range of time participants wore their Fitbit device was between 5.5 and 23.5 hours (331.98 to 1408.27 minutes). Among individuals aged 55 years or older, this range was between 11 and 23.5 hours (661.28 to 1408.27 minutes). As the minimum wear time found for participants 55 years or older was 11 hours across all 3 trials, 100% of older adults had 10 hours or more of Fitbit daily wear time. In addition, most participants across all 3 trials (100/148; 74%) had valid wear time of ≥10 hours per day for 80% or more of the study duration. This suggests that not only was the average wear time high but that participants kept high levels of adherence throughout the trial duration. A large proportion of older adults (52/65; 80%) had valid wear time for their Fitbit device over the duration of the trial. Fitbit wearable device adherence was generally high across all 3 trials. The lowest average wear time was 19.7 hours (mean 1182.05, SD 245.53 minutes) in Trial #3. Participants in Trial #3 also had the lowest frequency of valid wear time for 80% or more of the study (32/57; 56%). However, individuals who were 55 years or older had a greater frequency of Fitbit wear time for 80% or more of the study duration (11/14; 79%). Full results for Fitbit wearable device adherence can be found in Table 2.

**Table 2:** Fitbit activity tracker adherence.

Trial and group			Daily Fitbit wear time (minutes), mean (SD)		*P* value^a^		Daily Fitbit wear time (minutes), range		Sample with ≥10 hours of wear time, n (%)		*P* value^b^		Sample with valid wear time for ≥80% of the study, n (%)		*P* value^a^
**All trials**					.25						.13				.16
	Overall (n=148)	1217.47 (217.53)				331.98-1408.27		145 (98)				110 (74)			
	<55 (n=83)	1199.08 (229.12)				331.98-1408.09		80 (96)				58 (70)			
	55+ (n=65)	1240.94 (200.43)				661.28-1408.27		65 (100)				52 (80)			
**Trial #1**					.45						N/A^c^				.08
	Overall (n=59)	1263.73 (193.62)				661.28-1408.27		59 (100)				53 (90)			
	<55 (n=30)	1282.77 (163.37)				819.59-1408.09		30 (100)				29 (97)			
	55+ (n=29)	1244.04 (221.85)				661.28-1408.27		29 (100)				24 (83)			
**Trial #2**					.16										.86
	Overall (n=32)	1195.24 (196.08)				587.36-1386.87		31 (97)		.16		25 (78)			
	<55 (n=10)	1122.79 (231.17)				587.36-1374.85		9 (90)				8 (80)			
	55+ (n=22)	1228.17 (173.84)				817.05-1386.87		22 (100)				17 (77)			
**Trial #3**					.21						.43				.05
	Overall (n=57)	1182.05 (245.53)				331.98-1406.87		55 (96)				32 (56)			
	<55 (n=43)	1158.44 (253.61)				331.98-1406.59		41 (95)				21 (49)			
	55+ (n=14)	1254.58 (207.33)				709.31-1405.24		14 (100)				11 (79)			

^a^*P* values calculated using chi-square tests for continuous variables.

^b^*P* values calculated using the nonparametric Mann-Whitney *U* test due to zero-value cells which would violate assumptions of the chi-square test.

^c^Not applicable.

### Acceptability

In Trial #1, participants reported consistently high satisfaction levels with the Fitbit device (≥55: mean 5.36, SD 0.83; <55: mean 5.31, SD 0.97; *P*=.83), the information videos (≥55: mean 4.24, SD 1.27; <55: mean 3.96, SD 1.37; *P*=.42), daily SMS text message surveys (≥55: mean 4.45, SD 1.59; <55: mean 4.76, SD 1.38; *P*=.43), and not finding the study cumbersome to participate in (≥55: mean 4.66, SD 1.40; <55: mean 4.55, SD 1.48; *P*=.77). Notably, participants’ satisfaction with the digital onboarding process was slightly lower than other measures of satisfaction in Trial #1 (≥55: mean 3.89, SD 2.01; <55: mean 3.27, SD 2.21; *P*=.27). Participant satisfaction ratings did not significantly differ by age group.

Trial #2 participants also reported relatively high levels of satisfaction across measures, including with the Fitbit device (≥55: mean 4.44, SD 1.79; <55: mean 5.67, SD 0.52; *P*=.12), the digital onboarding process (≥55: mean 4.38, SD 1.93); <55: mean 5.20, SD 1.30; *P*=.35), the information videos (≥55: mean 4.25, SD 1.48; <55: mean 4.83, SD 1.83; *P*=.45), daily SMS text message surveys (≥55: mean 4.63, SD 1.31; <55: mean 4.5, SD 1.52; *P*=.84), and not finding the study cumbersome to participate in (≥55: mean 4.31, SD 1.74; <55: mean 4.33, SD 2.42; *P*=.98). It is important to note that only 22 out of 32 (69%) participants provided satisfaction measures in Trial #2 after the intervention completed. As with Trial #1, no significant differences in satisfaction ratings were found between age groups.

There were comparable results for Trial #3, with participants ≥55 years old endorsing high levels of satisfaction with the Fitbit device (≥55: mean 4.50, SD 0.67; <55: mean 4.48, SD 0.65; *P*=.93), the digital onboarding process (≥55: mean 4.75, SD 0.45; <55: mean 4.40, SD 0.58; *P*=.08), the information videos (≥55: mean 4.75, SD 0.45; <55: mean 4.24, SD 0.88; *P*=.07), daily SMS text message surveys (≥55: mean 4.08, SD 0.90; <55: mean 3.72, SD 1.10); *P*=.33), and not finding the study cumbersome to participate in (≥55: mean 4.75, SD 0.45; <55: mean 4.48, SD 0.77; *P*=.27). As in Trial #2, a smaller proportion of participants provided satisfaction data with only 37 out of 55 (67%) participants in Trial #3 completing the survey after the intervention completed. Full participant satisfaction results are reported in Table 3. No significant differences in satisfaction ratings by age group were found for Trial #3.

Across all 3 trials, the majority of participants expressed satisfaction with their Fitbit wearable device (Trial #1: 54/59, 92%; Trial #2: 18/22, 82%; Trial #3: 34/37, 92%). Compared to the full sample, individuals aged 55 years or older also expressed high levels of satisfaction with their Fitbit device (Trial #1: 27/29, 93%; Trial #2: 12/16, 75%; Trial #3: 11/12, 92%). The majority of participants were also satisfied with receiving SMS text message interventions and survey assessments (Trial #1 45/59, 76%; Trial #2 16/22, 73%; Trial #3: 23/37, 62%). The proportion of individuals aged 55 years and older who were satisfied with SMS text message interventions and surveys was also comparable to the full sample (Trial #1: 21/29, 72%; Trial #2: 11/16, 69%; Trial #3 8/12, 67%).

**Table 3:** Participant satisfaction with aspects of the personalized trials.

Satisfaction measure	Item scale	Trial #1				*P* value^a^		Item scale		Trial #2				*P* value^a^		Item scale		Trial #3				*P* value^a^
		All (n=59)	<55 (n=30)	55+ (n=29)					All (n=22)		<55 (n=6)	55+ (n=16)					All (n=37)		<55 (n=25)	55+ (n=12)		
My Fitbit device was easy to use, mean (SD)	0=Strongly disagree to 6=Strongly agree	5.33 (0.89)	5.31 (0.97)	5.36 (0.83)	.83		0=Strongly disagree to 6=Strongly agree		4.77 (1.63)		5.67 (0.52)	4.44 (1.79)	.12		1=Strongly disagree to 5=Strongly agree		4.49 (0.65)		4.48 (0.65)	4.50 (0.67)	.93	
I found the onboarding process trial straightforward and easy to follow, mean (SD)	0=Strongly disagree to 6=Strongly agree^b^	3.57 (2.12)	3.27 (2.21)	3.89 (2.01)	.27		0=Strongly disagree to 6=Strongly agree		4.57 (1.80)		5.20 (1.30)	4.38 (1.93)	.35		1=Strongly disagree to 5=Strongly agree		4.51 (0.56)		4.40 (0.58)	4.75 (0.45)	.08	
The informational videos helped me understand how to participate in this study, mean (SD)	0=Strongly disagree to 6=Strongly agree	4.11 (1.32)	3.96 (1.37)	4.24 (1.27)	.42		0=Strongly disagree to 6=Strongly agree		4.41 (1.56)		4.83 (1.83)	4.25 (1.48)	.45		1=Strongly disagree to 5=Strongly agree		4.41 (0.80)		4.24 (0.88)	4.75 (0.45)	.07	
I enjoyed receiving daily text message prompts and surveys on my cell phone, mean (SD)	0=Strongly disagree to 6=Strongly agree	4.60 (1.49)	4.76 (1.38)	4.45 (1.59)	.43		0=Strongly disagree to 6=Strongly agree		4.59 (1.33)		4.50 (1.52)	4.63 (1.31)	.84		1=Strongly disagree to 5=Strongly agree		3.84 (1.04)		3.72 (1.10)	4.08 (0.90)	.33	
I did not find my personalized trial to burdensome or cumbersome, mean (SD)	0=Strongly disagree to 6=Strongly agree^b^	4.60 (1.43)	4.55 (1.48)	4.66 (1.40)	.77		0=Strongly agree to 6=Strongly disagree^b^		4.32 (1.89)		4.33 (2.42)	4.31 (1.74)	.98		1=Strongly disagree to 5=Strongly agree		4.57 (0.69)		4.48 (0.77)	4.75 (0.45)	.27	
I would strongly recommend this trial, n (%)	N/A^c^	39 (65)	20 (51)	19 (49)	.93		N/A		N/A		N/A	N/A	N/A		N/A		35 (95)		23 (92)	12 (100)	.82	

^a^*P* values were generated using 2-tailed independent samples *t* tests for continuous variables and chi-square tests for categorical variables.

^b^This item was reverse scored in the initial measure but was recoded for ease of interpretation in this table.

^c^Not applicable.

## Discussion

### Principal Findings

These results suggest that personalized trials using digital recruitment strategies, remote intervention delivery, wearable activity trackers, and SMS text message–based surveys can be conducted among older adults, with high rates of feasibility and acceptability, similar to what is seen in younger adults. High adherence to Fitbit wearable activity trackers over time was seen across all trials and among older adults. It should be noted that all 3 trials encouraged participants to wear their Fitbits for 24 hours a day. Some trials only encourage Fitbit use during waking hours or during specific periods of time [60], perhaps explaining the higher levels of Fitbit device adherence in the current trial. Further, participants reported high levels of satisfaction with multiple aspects of the trial, including digital onboarding, the use of digital videos to describe the trial design, survey collection using SMS text messages, and reported low levels of burden from the personalized trial. Wearable device use and satisfaction were also comparable between older and younger adults in all 3 trials. These preliminary findings provide additional support for the use of these types of designs.

Digital, personalized trials offer a promising framework for providing accessible care, particularly to those who historically have experienced barriers based on geographical location, comorbidities, or complex risk factors. In general, personalized trials show great levels of promise for identifying the best intervention at the individual level and delivering personalized care [25,61]. Among aging adults with chronic conditions, telehealth interventions have been shown to improve self-care skills, self-monitoring behaviors, and clinical outcomes [62]. Remote interventions, such as those offered by telehealth, continue to promote self-care, which empowers patients and promotes long-term wellness [63]. In higher-need populations, such as individuals with serious mental illness and chronic health conditions, remote psychoeducational interventions improved participants’ understanding of their health condition [64]. However, though personalized trials may generally be effective in older adults, the method in which the intervention is operationalized can affect ratings of feasibility and acceptability. The content, method of delivery, and frequency of interventions can all influence participant ratings of feasibility and acceptability [32,65,66]. With careful consideration of the population of interest, personalized trials are one of the most powerful tools available to us in the precision-medicine era [67], particularly when using digital, remote interventions [68].

Digital, personalized trials offer a promising method of providing accessible opportunities for older adults to receive remote interventions and to have the effectiveness of those interventions assessed digitally. This model can continue to promote intervention delivery while building the research knowledge base for the older adult population despite interruptions to daily life like the COVID-19 pandemic. Given the great potential of the digital, personalized field of research, further implications regarding service delivery are worth exploring.

With the successful implementation of digital, personalized trials among older adults, there is an opportunity to integrate these remote trials into the clinical encounter to best identify which treatments may work best for a unique individual [25]. This integration has many benefits, including allowing older adult patients to establish themselves as a key stakeholder in the development of their treatment plans with their providers, paving the way for greater confidence in self-managed care without sacrificing quality data collection, and reducing the burden of labor and cost for patients, providers, and insurance companies.

Our results are not the first to demonstrate the feasibility and acceptability of digital, remote trials. Such evidence has been found among several clinical populations, including those with cardiovascular disease [69], depression [70], kidney transplants [71], telehealth services for veterans [72], and chronic conditions [73]. Our results extend these findings by including aging adults as the population of interest and by including personalization as an additional trial component. Furthermore, our findings suggesting participant satisfaction with video-based trial education are some components of our discussed findings that may present opportunities for other use cases, such as video-based education to encourage clinical trial enrollment [74], educate participants on their chronic illnesses [75], and even increase representation of diverse populations [76]. While in-person care will continue to be a critical aspect of clinical treatment, remote trials serve to demonstrate the feasibility of personalized trials and integration of personalized trials into clinical care can low-cost, low-burden, and high-access care provision in cases when in-person care is not required or available.

### Limitations

Although preliminary evidence from 3 innovative trials indicates that older adults may effectively engage with and be satisfied with a digital, remote, personalized trial design, there are several important limitations to note. First, the personalized trials discussed in this study included older adults but were not all specifically designed for older adults. Future trials tailored specifically to older adults may show even better results. Second, a number of participants in Trial #2 (10/32; 32%) and Trial #3 (18/55; 33%) did not complete the satisfaction survey at the end of the trial. This may limit the interpretability of these satisfaction reports. However, no differences were found in survey completion based on participant demographics [41,77]. Third, the reliance on effective technology to deliver digital, personalized interventions in all 3 trials may limit the use of these designs in areas with poor cellular connections or limited internet access. Fourth, our trials used age ≥55 to define older age. This was to more broadly capture older adults who may have difficulty interacting with technology. However, this may also limit the strengths of applying our findings to older individuals. The current analyses did not compare age groups among older adults (eg, 55 to 64 years old, 65 to 74 years old, and 75 years and older). Small sample sizes of older adults in each trial (<30 participants per trial) made comparisons of device use and satisfaction between older age groups difficult but we intend to continue pooling data from personalized trials to make these comparisons in the future. Finally, we acknowledge that cohort factors, including experience with digital devices, may have differentially influenced participants’ ease of device integration into daily life. Though we did not assess participants on their experience with digital devices in this study, such a metric may be useful for future investigations into this topic.

### Conclusions

The results from this study reveal the feasibility and acceptability of digital, remote personalized trials for adults older than 55 years by demonstrating good satisfaction and adherence. High levels of satisfaction and adherence among adults using digital resources for trial participation in the context of the COVID-19 pandemic suggests that digital, remote delivery of personalized trials is not only possible but may even provide greater benefit via flexibility to trial participants. To enable future digital personalized trials across health care, advancements in the following domains will be necessary: access to high-quality internet or cell phone service and private spaces, software usability, digital literacy, and legal considerations of collecting data and delivering interventions remotely across the United States [78]. Fortunately, the promise of digital, personalized service delivery appears to far outweigh the challenges of technological integration into medicine.

## Appendix

Multimedia Appendix 1 Trial eligibility criteria.

## Abbreviations

BCT: behavior change technique
CLBP: chronic lower back pain
CONSERVE: CONSORT and SPIRIT Extension for RCTs Revised in Extenuating Circumstances
CONSORT: Consolidated Standards of Reporting Trials
RCT: randomized controlled trial
SPIRIT: Standard Protocol Items: Recommendations for Interventional Trials
