# Supplementary material for: Digital, Personalized Clinical Trials Among Older Adults, Lessons Learned From the COVID-19 Pandemic, and Directions for the Future: Aggregated Feasibility Data From Three Trials Among Older Adults
Source: J Med Internet Res. 2025 Apr 16;27:e54629. doi: 10.2196/54629 (PMC12044319; doi:10.2196/54629)
Supplement: Multimedia Appendix 1 [file jmir_v27i1e54629_app1.docx]

| Trial #1 – ***Behavior Change Techniques (BCTs) to Improve Low Intensity Physical Activity in Older Adults*** | Trial #2 – ***A Trial of Habit Formation Theory for Exercise in Older Adults*** | Trial #3 – ***Personalized Trial for Chronic Lower Back Pain*** |
| --- | --- | --- |
| - Age 45 - 75 years old of age - Fluent in English - Employed in the Northwell Health system - Community-dwelling - Report they are in good general health, walk regularly and have never been informed by a clinician that it was not advisable/safe to participate in a low-intensity walking program - Owns and can regularly access a smartphone capable of receiving text messages - Owns and can regularly access an e-mail account | - Age 45 - 75 years old of age - Fluent in English - Employed in the Northwell Health system - Community-dwelling - Report they are in good general health, walk regularly and have never been informed by a clinician that it was not advisable/safe to participate in a low-intensity walking program - Owns and can regularly access a smartphone capable of receiving text messages - Owns and can regularly access an e-mail account | - At least 18 years of age - Fluent in English - Experiencing symptoms of lower back pain for 12 or more weeks - Experiencing a self-reported pain intensity ≥ eight on the Patient-Reported Outcomes Measurement Information System (PROMIS) Pain Interference 8a short-form scale - Able to receive interventions (two times per week between 8:00 a.m. and 10:00 p.m. Monday through Sunday) - Possessing a smartphone capable of receiving text messages - Possessing an e-mail account that can be regularly accessed |
